# Assessment of degenerative cervical myelopathy differs between specialists and may influence time to diagnosis and clinical outcomes

**DOI:** 10.1371/journal.pone.0207709

**Published:** 2018-12-17

**Authors:** Bryn Hilton, Jennifer Tempest-Mitchell, Benjamin Davies, Mark Kotter

**Affiliations:** 1 School of Clinical Medicine, University of Cambridge, Cambridge, United Kingdom; 2 Academic Neurosurgery Unit, Department of Clinical Neurosurgery, University of Cambridge, Cambridge, United Kingdom; University of Utah Hospital, UNITED STATES

## Abstract

**Introduction:**

Degenerative Cervical Myelopathy [DCM] often presents with non-specific symptoms and signs. It progresses insidiously and leads to permanent neurological dysfunction. Decompressive surgery can halt disease progression, however significant delays in diagnosis result in increased disability and limit recovery. The nature of early DCM symptoms is unknown, moreover it has been suggested incomplete examination contributes to missed diagnosis. This study examines how DCM is currently assessed, if assessment differs between stages of healthcare, and whether this influences patient management.

**Study design:**

Retrospective cohort study.

**Methods:**

Cervical MRI scans (N = 1123) at a tertiary neurosciences center, over a single year, were screened for patients with DCM (N = 43). Signs, symptoms, and disease severity of DCM were extracted from patient records. Patients were considered at 3 phases of clinical assessment: primary care, secondary care, and surgical assessment.

**Results:**

Upper limb paraesthesia and urinary dysfunction were consistently the most and least prevalent symptoms respectively. Differences between assessing clinicians were present in the reporting of: limb pain (p<0.005), objective limb weakness (p = 0.01), hyperreflexia (p<0.005), Hoffmann reflex (p<0.005), extensor plantar reflex (p = 0.007), and lower limb spasticity (p<0.005). Pathological reflexes were least frequently assessed by primary care doctors.

**Conclusion:**

DCM assessment varies significantly between assessors. Reporting of key features of DCM is especially low in primary care. Incomplete assessment may hinder early diagnosis and referral to spinal surgery.

## Introduction

Degenerative Cervical Myelopathy (DCM) is characterized by cervical spinal cord compression secondary to degenerative pathologies of the cervical spine, including osteophytosis, intervertebral disc protrusion, and ligament hypertrophy or ossification [1]. It is the most common cause of spinal cord impairment worldwide and its association with advancing age makes it a pertinent issue in the aging population [1–3]. DCM leads to progressive neurological dysfunction causing symptoms such as upper limb weakness and sensory dysfunction, lower limb weakness and spasticity leading to imbalance and falls, sphincter dysfunction progressing to incontinence, as well as neck and limb pain [4–7]. DCM can cause severe functional disability for patients and lead to marked reduction in quality of life [8,9].

DCM is diagnosed by MRI evidence of cervical spinal cord compression in the context of clinical myelopathy [2,10]. Decompressive surgery remains the mainstay of treatment for DCM. The aim of surgery is to halt disease progression but it can also offer some meaningful symptomatic benefit to patients [11–13]. International guidelines advise that all patients with moderate or severe DCM should be offered surgery, as well as any patients with progressive disease [14]. Better post-operative functional outcomes are achieved when surgery takes place early when symptoms are still limited, due to the limited regenerative capacity of the spinal cord [15–17].

Delay in diagnosis is an established problem in DCM [18]; patients frequently wait years before receiving a diagnosis of DCM and this contributes to incomplete recovery and lifelong disability. In a progressive disease, such as DCM, this leads to increased disability and limits post-operative functional improvement. The reasons for this delay are multifactorial [19,20], but it is thought incomplete assessments are an important factor. The challenge for assessment is that symptoms appear variable and non-specific. Examination findings in isolation have limited predictive power and MRI image findings compatible with DCM can in fact be incidental [21,22]. Moreover the order in which symptoms and signs typically develop in DCM is not known, thus adding to the difficulty in catching DCM early before it leads to neurological deterioration.

The objective of this study was to consider how DCM is currently assessed, if assessment differs between specialties or stages of healthcare, and whether this influences patient management.

## Method

### Cohort formation

**Fig 1:**
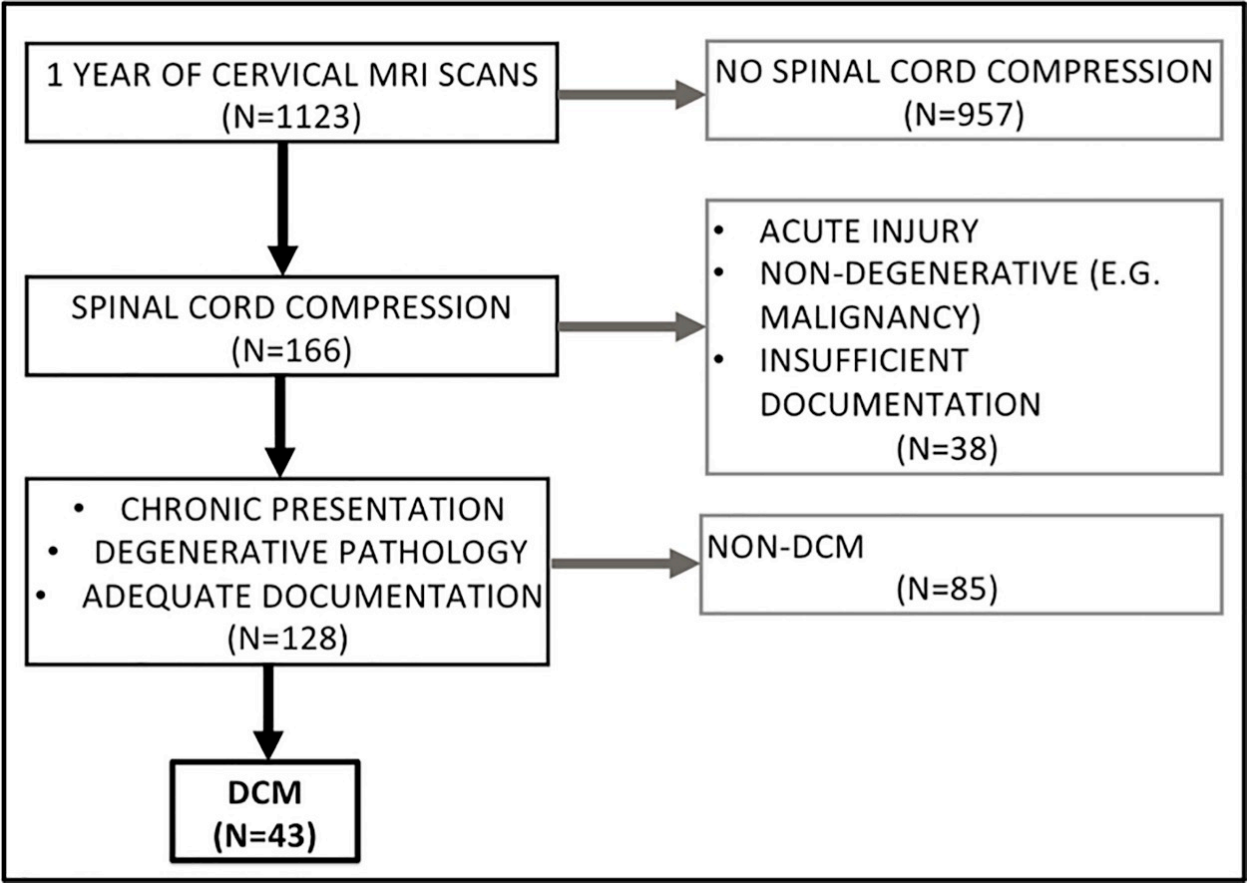
PRISMA flow diagram depicting cohort formation methodology.

One year of cervical MRI scans conducted at a tertiary neurosciences center were retrospectively collected and their reports were screened for identification of cord compression (Fig 1). Those scans that described cord compression in their reports, regardless of the degree of cord compression, were taken forward to the next step of cohort formation. All of the patient’s clinical records of each of these scans showing compression were subsequently screened for two inclusion criteria. Firstly, a clinical diagnosis of DCM. And secondly, sufficient documentation available to characterize referral pathway and clinical assessments. Cases that fit all of these criteria were fully anonymised and included in the cohort (N = 43). Characteristics of DCM, including demographics and disease severity were extracted from patient records. A tool was developed and prospectively validated to assess disease severity based on the mJOA. This is termed the ‘inferred’ mJOA or i-mJOA [23].

### Characterizing clinician assessment

The presence or absence of symptoms and signs consistent with DCM were adapted from Karpova et al 2014 [24]. Symptoms recorded were limb paraesthesia, subjective limb weakness, limb pain, neck pain/stiffness, sphincter dysfunction, imbalance, falls. Signs recorded were objective limb weakness, hyperreflexia, positive Hoffmann reflex, extensor plantar reflex, clonus, unstable gait, lower limb spasticity, and atrophy of the intrinsic muscles of the hand.

Each symptom and sign was recorded as present (P), absent (A), or undocumented (U). Data was grouped into three phases of clinical care: primary care (general practitioner or community physiotherapist), secondary care (first specialist appointment) and surgical (first assessment by a spinal surgeon).

### Analysis

Statistical analysis was carried out using SPSS V22 (Chicago, IL, USA). Bivariate analysis was conducted between variables using Pearson’s and Spearman’s correlations depending on the variable types involved. Chi-squared test of homogeneity was used to assess differences in reporting signs and symptoms between primary, secondary, and surgical assessments. Subsequent post hoc analysis involved pairwise comparisons using the z-test of two proportions with a Bonferroni correction to determine specific group differences in clinical feature reporting.

## Results

### Primary assessment

**Fig 2:**
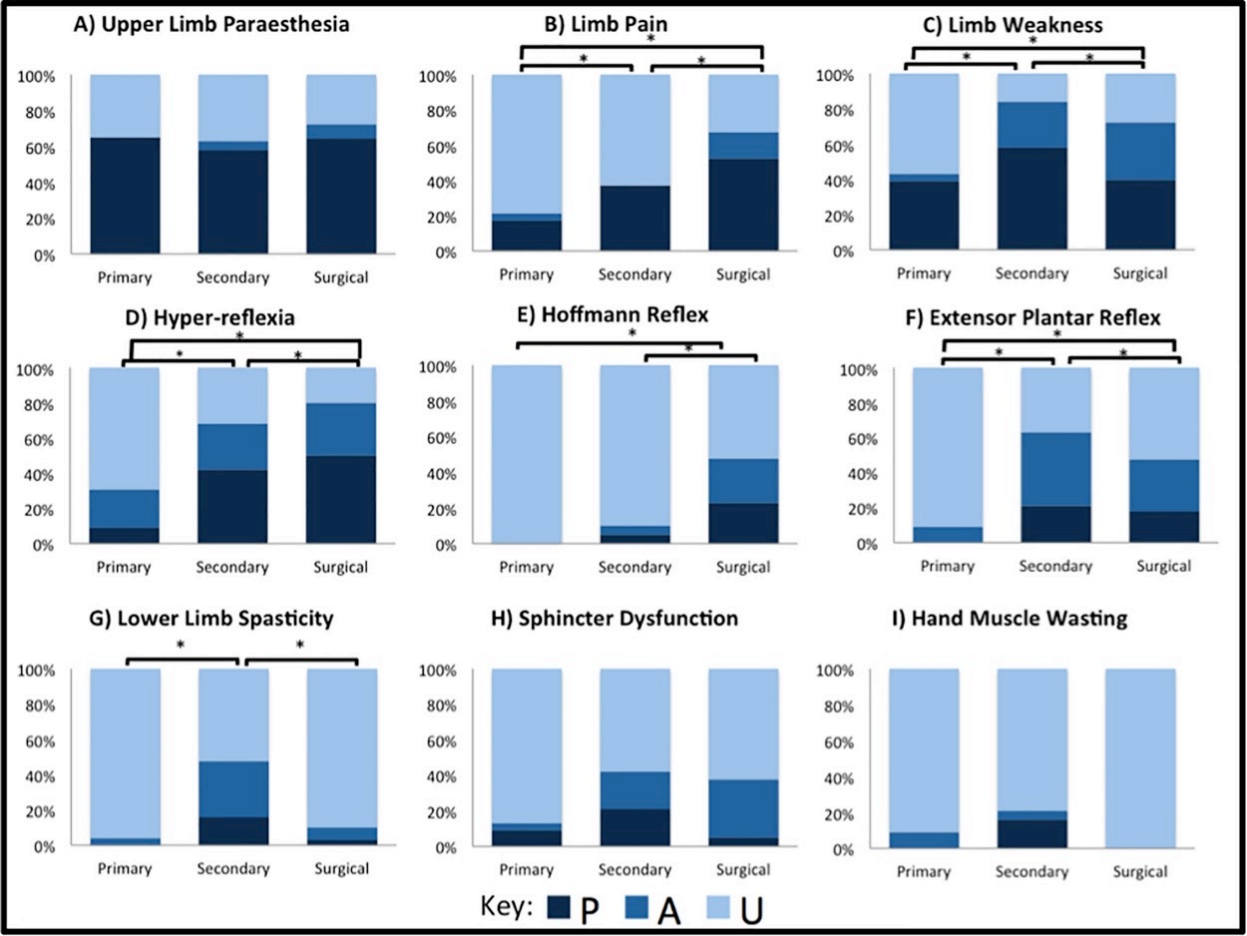
Differences in assessment of DCM patients. (A) Upper limb paraesthesia. (B) Limb pain. (C) Limb weakness (objective). (D) Hyperreflexia. (E) Hoffmann reflex. (F) Extensor plantar reflex. (G) Lower limb spasticity. (H) Sphincter dysfunction. (I) Hand muscle wasting. * denotes p<0.05.

**Fig 3:**
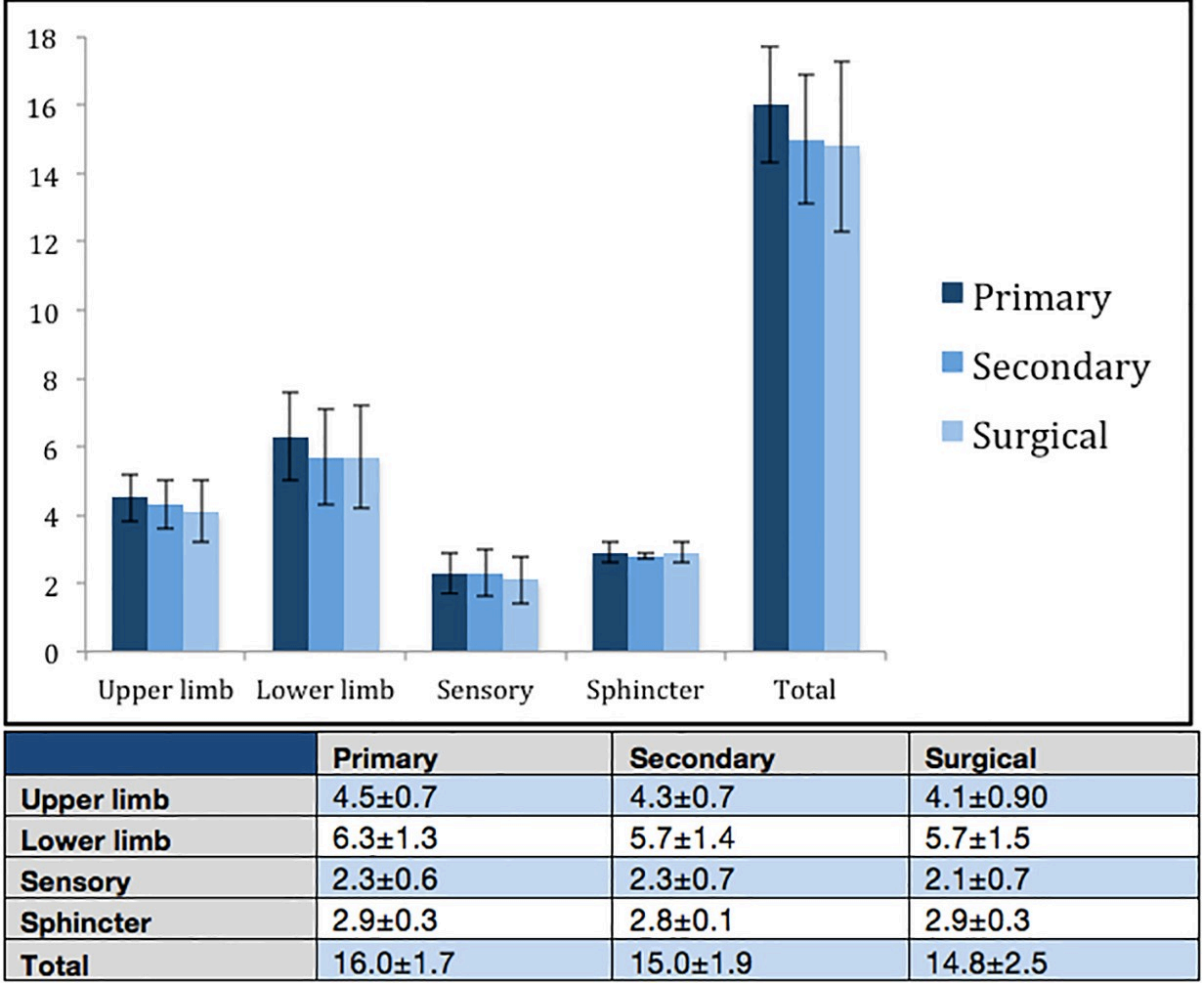
i-mJOA scores at each clinical phase of assessment. Mean ± standard deviation.

The details of 23 primary assessments were accessible. Upper limb paraesthesia was the most commonly reported symptom by patients (65%, N = 15), followed by lower limb weakness (43%, N = 10), and neck pain (43%, N = 10) (Fig 2). Objective limb weakness (upper or lower limb) was the most commonly detected feature on clinical examination (39%, N = 9). It was not possible to always distinguish upper limb weakness from lower limb weakness in reported clinical examination due to lack of clarity in assessment documentation. Sphincter function was only commented on in 13% of referrals (N = 3). Pathological reflexes besides hyperreflexia were seldom mentioned in primary referrals: hyperreflexia (41%), extensor plantar reflex (9%), clonus (4%), and positive Hoffman reflex (0%). i-mJOA at primary assessment was: upper limb 4.5±0.7, lower limb 6.3±1.3, sensory 2.3±0.6, sphincter 2.9±0.3, total 16.0±1.7 (Fig 3).

### Secondary assessment

The details of 19 secondary assessments were accessible. The most commonly documented symptoms were: upper limb paraesthesia (58%, N = 11), lower limb weakness (42%, N = 8), neck pain (42%, N = 8), and limb pain (37%, N = 7) (Fig 2). Clinical examination documented limb weakness (58%) and hyperreflexia (42%) as the most prominent features. Other pathological reflexes were commented on more frequently than in primary assessment: extensor plantar reflex (37%), clonus (21%), and positive Hoffman reflex (10%). i-mJOA at secondary assessment was: upper limb 4.3±0.7, lower limb 5.7±1.4, sensory 2.3±0.7, sphincter 2.8±0.1, total 15.0±1.9 (Fig 3).

### Surgical assessment

The details of 40 assessments made by spinal surgeons were accessible. The most prominent symptoms recorded during this assessment were upper limb paraesthesia (65%, N = 26) and limb pain (53%, N = 21) (Fig 2). Other notable symptoms documented included upper limb weakness (38%, N = 15), lower limb paraesthesia (38%, N = 15), neck pain (35%, N = 14), and lower limb weakness (33%, N = 13). Subjective imbalance was documented in 25% of patients (N = 10). Clinical examination revealed limb weakness in 40% of patients. Pathological reflexes were commented on most frequently in surgical assessments: hyperreflexia (80%), Hoffmann reflex (47%), extensor plantar reflex (47%), and clonus (16%). An unstable gait was noted in 28% of patients. i-mJOA at surgical assessment was: upper limb 4.1±0.9, lower limb 5.7±1.5, sensory 2.1±0.7, sphincter 2.9±0.3, total 14.8±2.5 (Fig 3).

### Differences in assessment

Differences were present in the reporting of: limb pain (p<0.005, Fig 2B), objective limb weakness (p = 0.01, Fig 2C), hyperreflexia (p<0.005, Fig 2D), Hoffmann reflex (p<0.005, Fig 2E), extensor plantar reflex (p = 0.007, Fig 2F), and lower limb spasticity (p<0.005, Fig 2G). The reporting of sphincter dysfunction (p = 0.072, Fig 2H) and intrinsic hand muscle wasting (p = 0.058, Fig 2I) was not significantly different. Post hoc analysis revealed limb weakness and extensor plantar reflex were more commonly not documented in primary assessment (p<0.05) whilst upper limb pain, hyperreflexia, and lower limb spasticity were more commonly documented during secondary assessment (p<0.05). The Hoffmann reflex was more commonly documented during surgical assessment (p<0.05).

Upper limb paraesthesia (Fig 2A) and sphincter dysfunction (Fig 2H) were consistently the most and least prevalent symptoms respectively. Limb pain also varied between assessors with a trend towards more patients reporting pain by surgical assessment. Imbalance and falls showed a consistent relationship regardless of assessor. Approximately half of patients who describe subjective imbalance also report falls.

The clinical assessment of pathological reflexes also differed between assessors. Hyperreflexia and extensor plantar reflexes were commonly assessed by secondary care doctors and spinal surgeons but rarely by primary care doctors. Clonus showed low levels of reporting at secondary and surgical assessments, 21% and 26% respectively, although again lowest in the community setting at only 4%. Furthermore, it was exceedingly rare for any clinician besides a spinal surgeon to assess the Hoffmann reflex.

### Impact of assessment on patient flow

Factors associated with a shorter time between primary assessment and secondary assessment were: subjective imbalance (r = -0.83, p = 0.04), i-mJOA lower limb (r_s_ = -0.47, p = 0.03), and age at time of onset (r = -0.48, p = 0.02). No clinical symptom or sign was associated with a significantly different speed of referral from secondary care to spinal surgeon review.

## Discussion

DCM is known to present with non-specific symptoms and signs [19,20]. Clinical signs such as objective weakness and pathological reflexes are not always present [24–27]. The order and number of clinical signs that develop in DCM with disease progression are currently unknown. It is likely that fewer clinical signs are present at early stages and mild disease than in severe disease. Incomplete examination may miss these [28]. The presence of neurological signs (e.g. hyperreflexia, extensor plantar reflex) is strongly indicative of an underlying pathology. Therefore, a thorough, targeted history and clinical examination are key to detecting DCM in its early stages.

If DCM, or another neurological condition, is suspected then a complete neurological examination should be conducted [2]. This should include formal upper and lower limb sensory, motor, and reflex assessment. Pathological reflexes are not always present in DCM at onset but become more common as the disease progresses [26,27,29]. Therefore, testing of a single reflex is insufficient to detect early DCM. However, a combination of Hoffmann’s reflex, extensor plantar reflex, and finger flexion reflex may be used as a rapid screening tool [30]. When tested together, these reflexes have been shown to have a sensitivity of 91.67% and specificity of 87.5% in determining if a patients symptoms are due to cervical spinal cord compression. As it is rapid and non-invasive, multiple reflex testing should be incorporated for clinical screening to guide investigation and triage, especially in primary care.

This study found differences in clinical feature reporting between primary, secondary, and surgical assessments. This may be due to disease progression, lack of documentation, or limited clinical examinations. We found low levels of reporting with respect to sphincter dysfunction at all phases of assessment (Fig 2H). As a key feature of DCM, sphincter dysfunction is important to enquire about. Furthermore, fewer clinical features were recorded in primary care overall (Fig 2). The limited extent of community neurological examinations has previously been noted [31]. The most common component of clinical examination observed in our study was objective limb weakness (Fig 2C). Primary care also showed very low levels of reporting on Hoffmann reflex and extensor planter reflex (Fig 2E and Fig 2F). “Neurophobia” in primary care, as well as more broadly in medicine, is a well-documented phenomenon and therefore these findings are unsurprising [32,33]. However, an incomplete clinical examination may hinder early detection and referral of DCM and thus introduces delays into the diagnostic pathway for DCM. Therefore, the multiple reflex testing represents a key area in which clinical evaluation can be improved.

This study found that subjective imbalance was the only symptom associated with a shorter referral time between primary and secondary assessment. However, i-mJOA lower limb and age at time of onset were also associated. This may be due to a drive to minimize falls risk [34] or due to ruling out a number of other pathologies requiring urgent assessment that may present with lower limb dysfunction in an older population (e.g. motor neuron disease [35]).

No clinical feature significantly influenced referral speed between secondary care and spinal surgeons. This indicates a lack of a streamlined pathway for urgent surgical assessment of patients with proven spinal cord compression and moderate or severe DCM or progressive disease, as per international guidelines [14]. Implementation of such a streamlined service would facilitate more rapid assessment for surgery and thus could improve patient outcomes using existing treatment strategies.

### Limitations

A potential issue with this study is a discrepancy between clinical examination and documentation. This limits conclusions regarding prevalence of DCM clinical features. However, as documented features should accord with what a clinician observed and deemed important, this study provides valuable insight into the scope of clinical assessment of DCM.

The epidemiology of DCM remains poorly characterized. However, DCM is becoming increasingly understood to be a more common condition than expected, especially given the current aging population. Although this study had a relatively low sample size of 43 cases of DCM, clear patterns of assessment differences did emerge. Furthermore, the low number of identified cases over one year potentially further implicates the difficulty in detecting DCM early in its disease course.

## Conclusions

DCM assessment varies significantly between clinician groups. Reporting of key features of DCM is especially low in primary care. Incomplete assessment may hinder early diagnosis and referral to spinal surgery. A more thorough clinical assessment could lead to early consideration of surgery and therefore better patient outcomes using existing treatment methods. One key area of change could be implementation of multiple reflex testing in primary care as a screening tool for DCM.

## Supporting information

S1 File This file contains the raw collected data used in this study. (XLSX)
